# A seven-autophagy-related gene signature for predicting the prognosis of differentiated thyroid carcinoma

**DOI:** 10.1186/s12957-022-02590-6

**Published:** 2022-04-23

**Authors:** Chengxin Li, Qianqian Yuan, Gaoran Xu, Qian Yang, Jinxuan Hou, Lewei Zheng, Gaosong Wu

**Affiliations:** Department of Breast & Thyroid Surgery, Zhongnan Hospital of Wuhan University, Wuhan, 430071 China

**Keywords:** Differentiated thyroid carcinoma, Autophagy-related genes, Prognostic risk model, Nomogram, Gene set enrichment analysis, The Cancer Genome Atlas

## Abstract

**Background:**

Numerous studies have implicated autophagy in the pathogenesis of thyroid carcinoma. This investigation aimed to establish an autophagy-related gene model and nomogram that can help predict the overall survival (OS) of patients with differentiated thyroid carcinoma (DTHCA).

**Methods:**

Clinical characteristics and RNA-seq expression data from TCGA (The Cancer Genome Atlas) were used in the study. We also downloaded autophagy-related genes (ARGs) from the Gene Set Enrichment Analysis website and the Human Autophagy Database. First, we assigned patients into training and testing groups. R software was applied to identify differentially expressed ARGs for further construction of a protein-protein interaction (PPI) network for gene functional analyses. A risk score-based prognostic risk model was subsequently developed using univariate Cox regression and LASSO-penalized Cox regression analyses. The model’s performance was verified using Kaplan-Meier (KM) survival analysis and ROC curve. Finally, a nomogram was constructed for clinical application in evaluating the patients with DTHCA. Finally, a 7-gene prognostic risk model was developed based on gene set enrichment analysis.

**Results:**

Overall, we identified 54 differentially expressed ARGs in patients with DTHCA. A new gene risk model based on 7-ARGs (CDKN2A, FGF7, CTSB, HAP1, DAPK2, DNAJB1, and ITPR1) was developed in the training group and validated in the testing group. The predictive accuracy of the model was reflected by the area under the ROC curve (AUC) values. Univariate and multivariate Cox regression analysis indicated that the model could independently predict the prognosis of patients with THCA. The constrained nomogram derived from the risk score and age also showed high prediction accuracy.

**Conclusions:**

Here, we developed a 7-ARG prognostic risk model and nomogram for differentiated thyroid carcinoma patients that can guide clinical decisions and individualized therapy.

**Supplementary Information:**

The online version contains supplementary material available at 10.1186/s12957-022-02590-6.

## Introduction

Autophagy involves autophagosome induction, phagophore nucleation and expansion, and lysosomal degradation [1, 2]. During cellular stress, autophagy is activated to maintain cellular homeostasis and energy balance [3]. Autophagy has been implicated in numerous cancer types [4] but its role in cancer is controversial. For instance, inhibition of autophagy may contribute to tumour growth and metastasis but promote chemotherapeutic sensitivity [5–7].

Thyroid cancer (THCA) is the most common endocrine malignancy [8]. Since the 1980s, advances in medical imaging technology and fine-needle aspiration technology for thyroid nodules have led to the detection of an increasing thyroid cancer incidence [9–11]. In 2017, the incidence of thyroid cancer in Korean women was 78.5/10^5^, second only to breast cancer among female cancers [12]. Based on pathological, clinical, and genetic features, malignant thyroid cancer can be classified into 5 subtypes: papillary, follicular, Hürthle cell, poorly differentiated, and anaplastic thyroid carcinoma [13]. Follicular thyroid carcinoma and papillary thyroid carcinoma are known as differentiated thyroid carcinomas and account for > 96% of thyroid carcinomas; these subtypes have good overall survival after standardized treatment with surgery, radioiodine ablation, and hormone therapy [14, 15]. However, distant metastasis, refractoriness to radioactive iodine, or locoregional recurrence significantly reduce overall DTHCA survival [16–18]. Numerous reports have implicated autophagy in thyroid carcinoma growth and treatment. For instance, inhibiting lactate dehydrogenase A (LDHA) activates autophagy via AMPK signalling, which has anti-cancer effects in papillary thyroid carcinoma [19]. Recent studies have shown that baicalein induces autophagy through ERK/PI3K/Akt signalling to suppress the growth of thyroid cancer cells [20]. Furthermore, autophagy can function as a therapeutic target for thyroid cancer treatment. For radioiodine (RAI)-refractory differentiated thyroid cancer, increasing the iodide uptake and concentration ability of thyroid follicular cells can significantly improve therapeutic efficacy [21]. Some researchers have focused on mediating the active iodine uptake function of the sodium iodide symporter (NIS), finding that autophagy-activating compounds activate the accumulation of intracellular Ca^2+^ and FOS, thus mediating the upregulation of NIS and restoration of RAI uptake [21, 22]. Wang et al. also found that inhibition of autophagy increased the sensitivity of BRAF-mutated thyroid cancer cells to the chemotherapeutic drug vemurafenib [23]. The above research results reflect the great potential of autophagic mechanism in the field of thyroid cancer treatment. The development of new autophagy-related diagnostic biomarkers may improve the early diagnosis and individualized treatment of DTHCA.

Here, autophagy-related genes (ARGs) were retrieved from the HADb database and GSEA website while THCA patients’ clinical features and gene expression data were downloaded from The Cancer Genome Atlas (TCGA). After differential expression analysis (between tumour and normal tissue) and functional enrichment analysis, prognosis-related genes were identified through Cox regression analyses. Next, a prognostic model was developed using LASSO-penalized COX regression analysis and its accuracy and independence validated. Finally, a prognostic nomogram for estimating patients’ overall survival based on independent clinical features and risk scores was constructed.

## Materials and methods

### Acquisition of ARGs

In total, 232 and 394 ARGs used in this study were acquired from the HADb database (http://www.autophagy.lu) and GO_AUTOPHAGY gene datasets from the Gene Set Enrichment Analysis (GSEA, http://www.gsea-msigdb.org/gsea/msigdb), respectively, as described previously [24]. Selection of ARGs that were common to the 2 datasets resulted in the identification of 531 ARGs for further analysis.

### Clinical features and gene expression data of THCA patients in TCGA

Clinical characteristics and transcriptome data were obtained from TCGA (https://portal.gdc.cancer.gov). In particular, the RNA-seq dataset contained data from 510 DTHCA tumour and 58 non-tumour tissues. The DTHCA patients were randomly assigned to the training group (*n* = 252) and testing group (*n* = 249) by random selection in SPSS (v.22.0). Patient data included age, gender, survival status, and pathological stage.

### Selection of differentially expressed autophagy-related genes

The Wilcoxon signed-rank test on R software (v.4.0.3) was employed to select differentially expressed autophagy-related genes (DEARGs). The cut-off criteria were | false discovery rate (FDR) < 0.05 and log_2_(fold change) | ≥ 1.

### Gene function analysis

To elucidate the DEARGs’ biological function, they were uploaded into Metascape (https://metascape.org). Subsequently, enrichment analysis based on Gene Ontology (GO) and Kyoto Encyclopedia of Genes and Genomes (KEGG) analyses was performed. The cut-off criteria were set as follows: *p*-value ≤ 0.05, Min Enrichment > 1.5, and Min Overlap = 3.

### Analysis of the PPI network

A PPI network was designed on the STRING v.11.0 database (https://www.string-db.org) and visualized with Cytoscape v.3.8.2 (https://cytoscape.org). MCODE v2.0, a Cytoscape plugin was used to identify densely connected modules (MCODE score ≥ 4.0, degree cut-off = 2).

### Establishment of an ARG prognostic model

The DEARG expression data were subjected to univariate Cox regression analysis to assess the associations of ARGs with OS (*p* ≤ 0.05 was applied as the inclusion criterion). RNA expression was normalized by log2 transformation. The hazard ratio (HR) was used to classify ARGs as protective or risk factors, with HR > 1 indicating protective factors and HR < 1 indicating risk factors. Based on LASSO-penalized Cox regression analysis, the prognosis-associated genes were used to develop a prognostic model. The risk score was the linear combination of gene expression and was calculated for each patient as follows: risk score=${\sum}_{i=1}^n\left( Coefi\ast Expi\right)$. *Coefi* stands for the regression coefficient of an ARG. *Expi* indicates the relative expression values of that ARG. The patients were then classified into the high- and low-risk groups using the median risk score of the training group as cutoff. Thereafter, Kaplan-Meier (KM) analysis and two-sided log-rank test were applied to compare OS between the two groups. The R package “timeROC” was used for ROC curve analysis to evaluate the prognostic model’s specificity and sensitivity. The ability of the prognostic risk model and clinical characteristics to independently predict OS was elucidated based on univariate and multivariate Cox regression analysis. Subgroup analysis was used to evaluate the model’s applicability.

### Multivariate ROC analysis

To compare the prognostic accuracy of our ARG signature to the TNM staging system, multivariate ROC analysis was performed by R package “timeROC”, “survival”, and “survminer”.

### Construction and validation of the nomogram

To enable clinicians to conveniently use the prognostic model in assessing the 3- and 5-year OS of patients with DTHCA, we used the independent risk factors (risk score and age) to create a nomogram and then validated its predictive ability by plotting calibration curves for comparing differences between predicted and actual survival. The Harrell concordance index (*C*-index) was used to assess the nomogram’s predictive accuracy.

### Gene set enrichment analysis

Gene set enrichment analysis (GSEA) for both the high- and low-risk groups was performed based on the C2.CP.KEGG.v7.4 gene set using GSEA software (version 4.1.0). The statistical significance criteria were set as NOM *p* < 0.05 and FDR < 0.25.

### Statistical analysis

Data analysis was performed using R version 4.0.3. KM analysis was performed using the R packages “survival” and “survminer”, and differences were assessed by the log-rank test. ROC curves were plotted using the R package “timeROC”. Univariate and multivariate Cox regression analysis was used to test the model’s prognostic independence. *p* ≤ 0.05 indicates statistical significance.

### Cell culture

The human normal thyroid epithelial cell line N-thy-ori-3-1 and the thyroid cancer cell lines FTC-133 and TPC-1 were obtained from Procell (Wuhan, China). All cell lines were cultured in DMEM (Irvine Scientific, Carlsbad, CA) supplemented with 10% foetal bovine serum (FBS).

### RNA extraction and qPCR analysis

RNA was extracted from the cell using HiPure Total RNA Mini Kit (R4111-03, Magen, China). HiScript II QRT SuperMix Kit (Vazyme, China) was used for reverse transcription. qRT-PCR was conducted using the SYBR GREEN MIX Kit (Vazyme, China) by the CFX96 Real-time PCR Detection System (Bio-Rad, USA). GAPDH was selected as the internal housekeeping gene, and relative gene expression was calculated by the 2^−ΔΔCt^ method. Each qRT-PCR was repeated three times.

## Results

### Identification of DEARGs in THCA patients

**Fig. 1:**
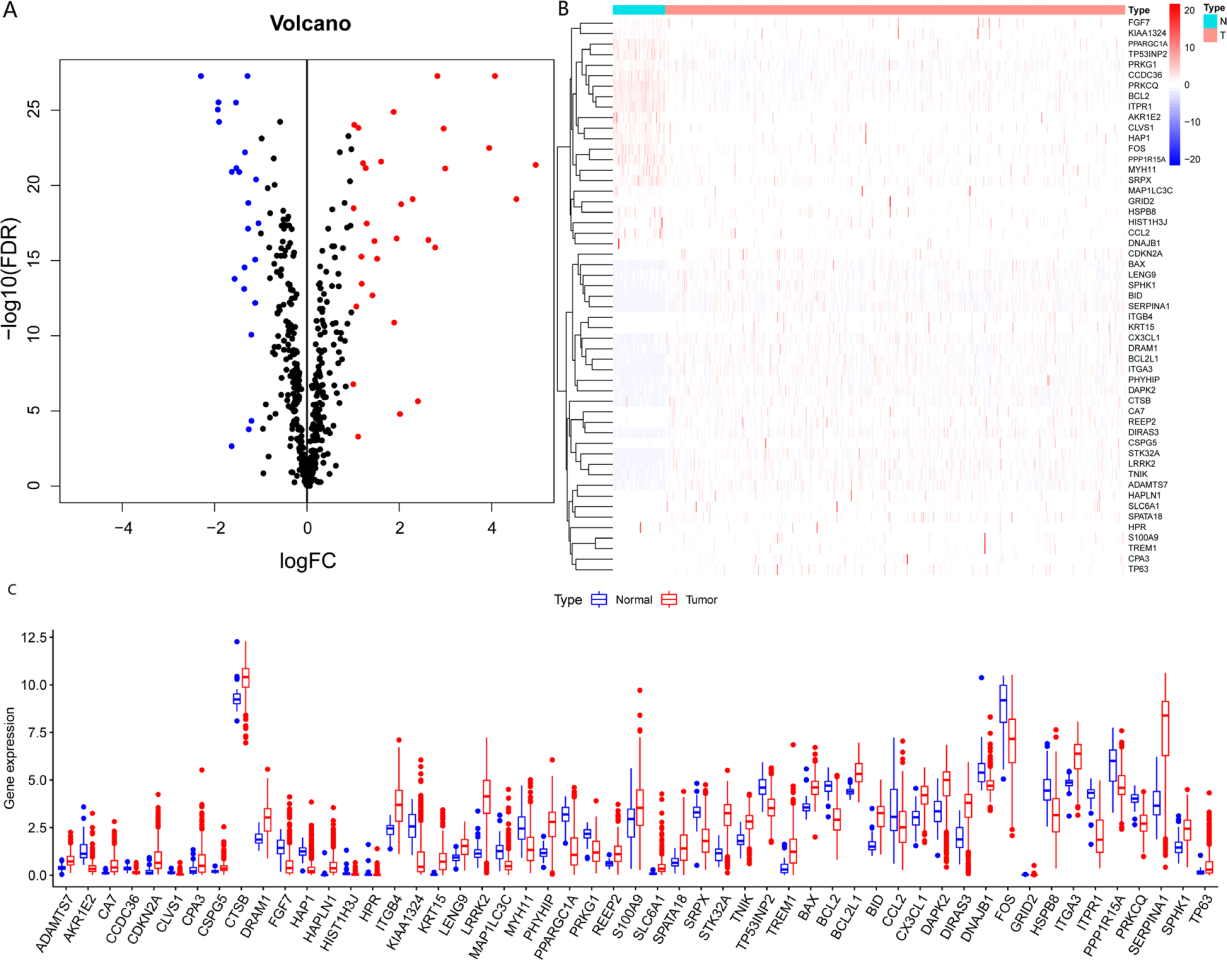
Differentially expressed autophagy related genes (DEARGs) between thyroid carcinoma tissues and non-tumour normal tissues. (**A**)Volcano plot for the DEARGs blue: downregulate red: upregulate. (**B**) Heatmap of 54 DEARGs. The depth of blue indicates the level of low expression, and the depth of red indicates the level of high expression. (**C**) Boxplot of the DEARGs

A total of 531 identified ARGs were used in the study (Table S1). Gene expression data and clinical information on 58 normal and 510 DTHCA tissues were downloaded from the TCGA database (Table 1). Application of the criteria FDR < 0.05 and | log_2_(FC) | ≥ 1 resulted in 54 DEARGs. Of these, 23 were downregulated and 31 were upregulated (Fig. 1A–C)

**Table 1:** Clinical characteristics of the TCGA-THCA dataset

Clinical characteristic	TCGA-THCA (*n* = 501)	Percentage
**Survival status**		
Alive	485	96.8%
Dead	16	3.2%
**Age**		
≥ 65	76	15.2%
< 65	425	84.8%
**Gender**		
Female	366	73.1%
Male	135	26.9%
**T**		
T1	142	28.3%
T2	164	32.7%
T3	170	33.9%
T4	23	4.6%
TX	2	0.5%
**N**		
N0	229	45.7%
N1	222	44.3%
NX	50	10.0%
**M**		
M0	282	55.3%
M1	9	1.8%
MX	210	42.9%
**AJCC stage**		
Stage I	281	56.1%
Stage II	52	10.4%
Stage III	111	22.2%
Stage IV	55	10.9%
Unknown	2	0.4%

### Functional annotation of DEARGs

**Fig. 2:**
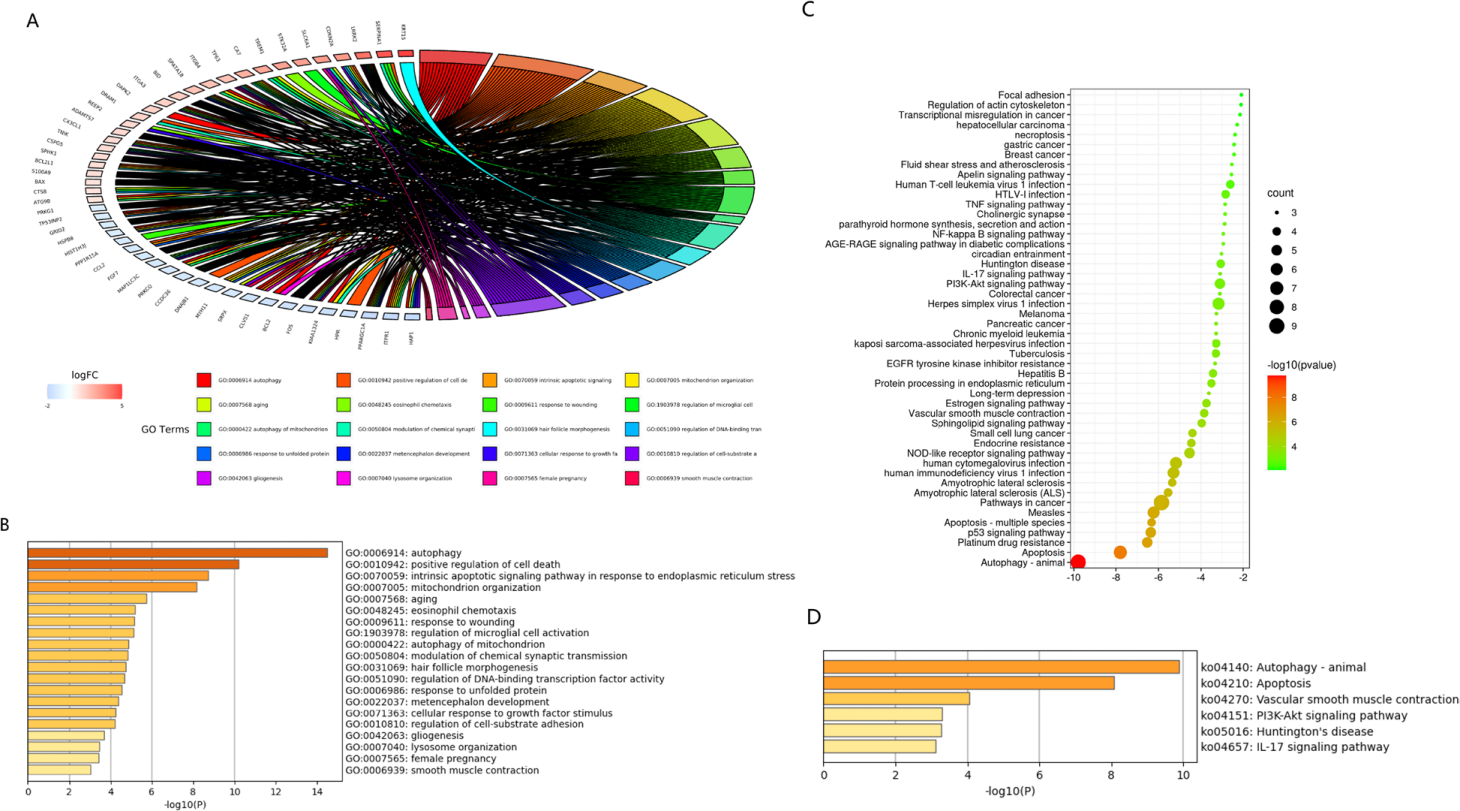
GO and KEGG functional enrichment analysis. (**A**) The circus plot of GO biological processes enrichment analysis. (**B**) Heatmap for the GO biological processes enrichment analysis. (**C**) The bubble plot of KEGG pathway enrichment analysis. (**D**) Heatmap for the KEGG pathway enrichment analysis

To elucidate the biological function and mechanisms of the 54 DEARGs, GO biological process and KEGG pathway enrichment analyses were done. The DEARGs showed strong enrichment in the GO terms autophagy, intrinsic apoptotic pathway in response to endoplasmic reticulum stress and positive regulation of cell death (Fig. 2A, B). KEGG pathway enrichment analysis revealed that the DEARGs were closely related to pathway autophagy-animal and apoptosis (Fig. 2C, D).

### Analysis of the PPI network

**Fig. 3:**
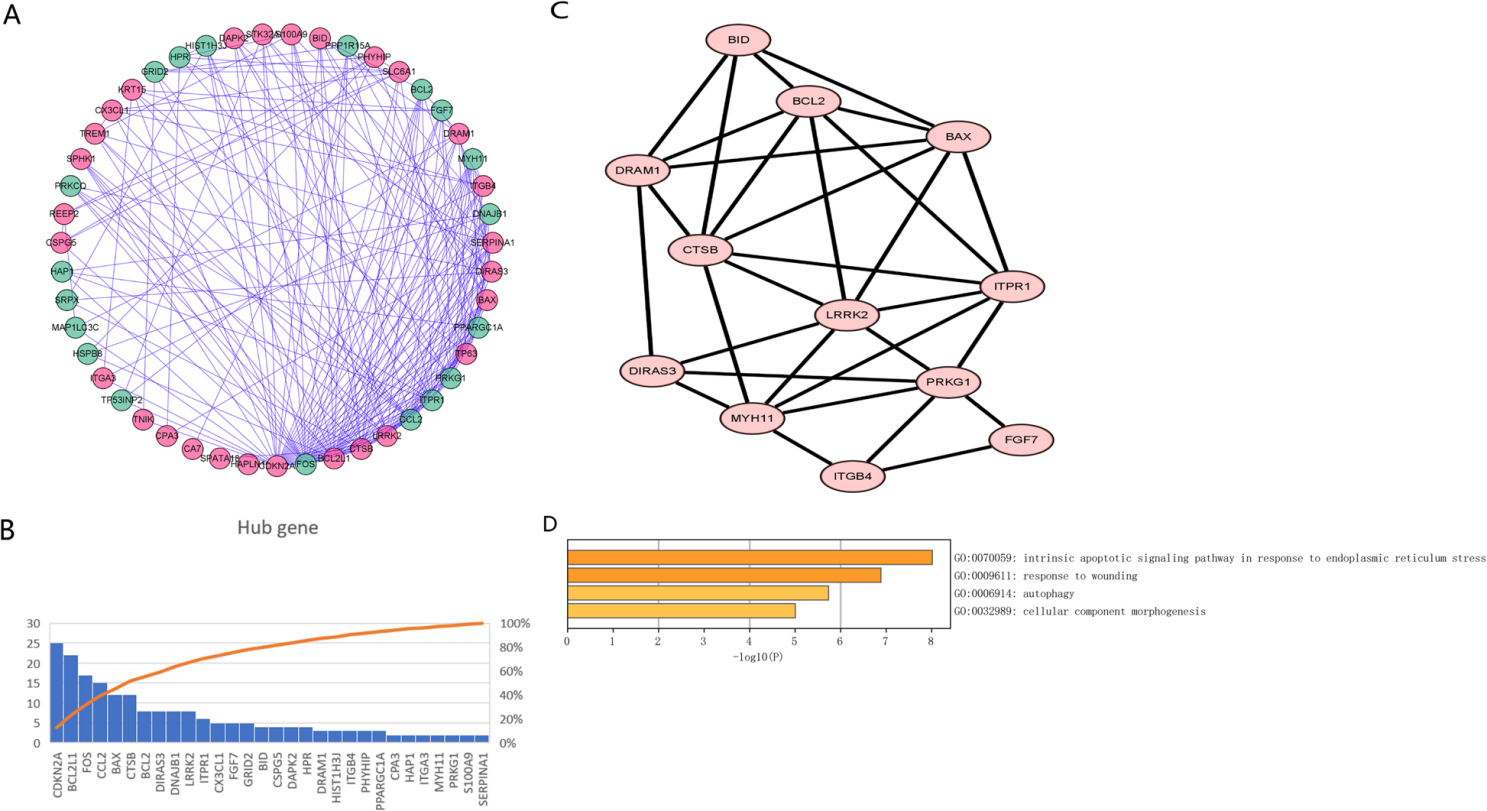
Protein-Protein Interaction (PPI) network analysis of DEARGs in THCA: (**A**) PPI network plot for DEAGRs Red: upregulate genes Green: downregulate genes. (**B**) Hub genes with more than 2 interactions. (**C**) Subnet modules of PPI network analysis. (**D**) GO biological processes enrichment analysis for the subnet modules

Examination of the PPI network of interaction between the DEARGs revealed 47 nodes and 208 edges (Fig. 3A; red = upregulation, green = downregulation). Next, 30 genes with > 2 edges were selected as the hub DEARGs for further analysis (Fig. 3B). Enrichment analysis of the module with the highest score revealed that the DEARGs in the module were associated with autophagy (Fig. 3C, D).

### Design of the ARG-based prognostic model

**Fig. 4:**
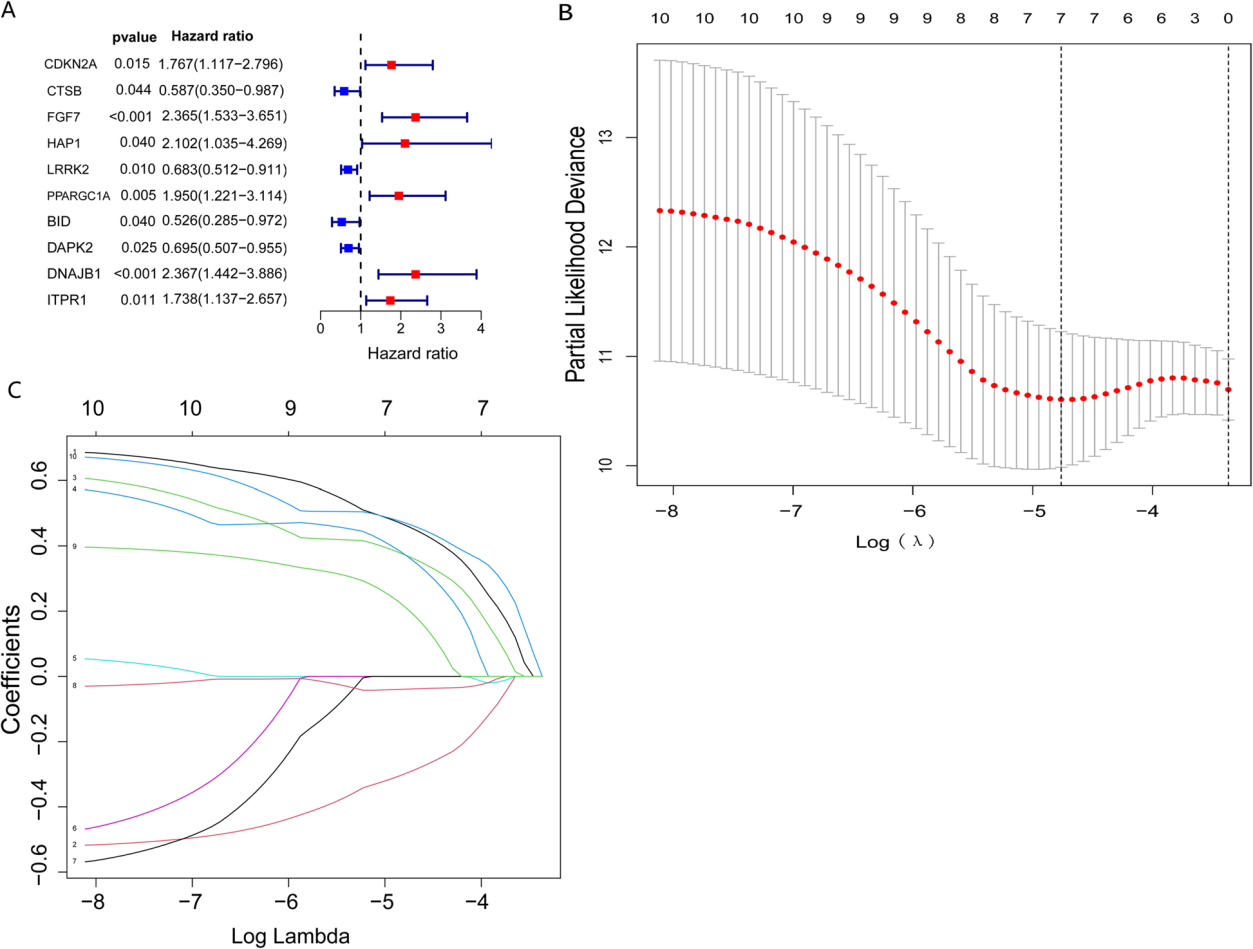
Key prognostic-related genes and construction of prognostic risk model: (**A**) Forest plot of 10 prognostic-related genes (**B**) Lasso Cox regression of 7 genes used in the prognostic risk model. (**C**) Lasso filters variables

**Fig. 5:**
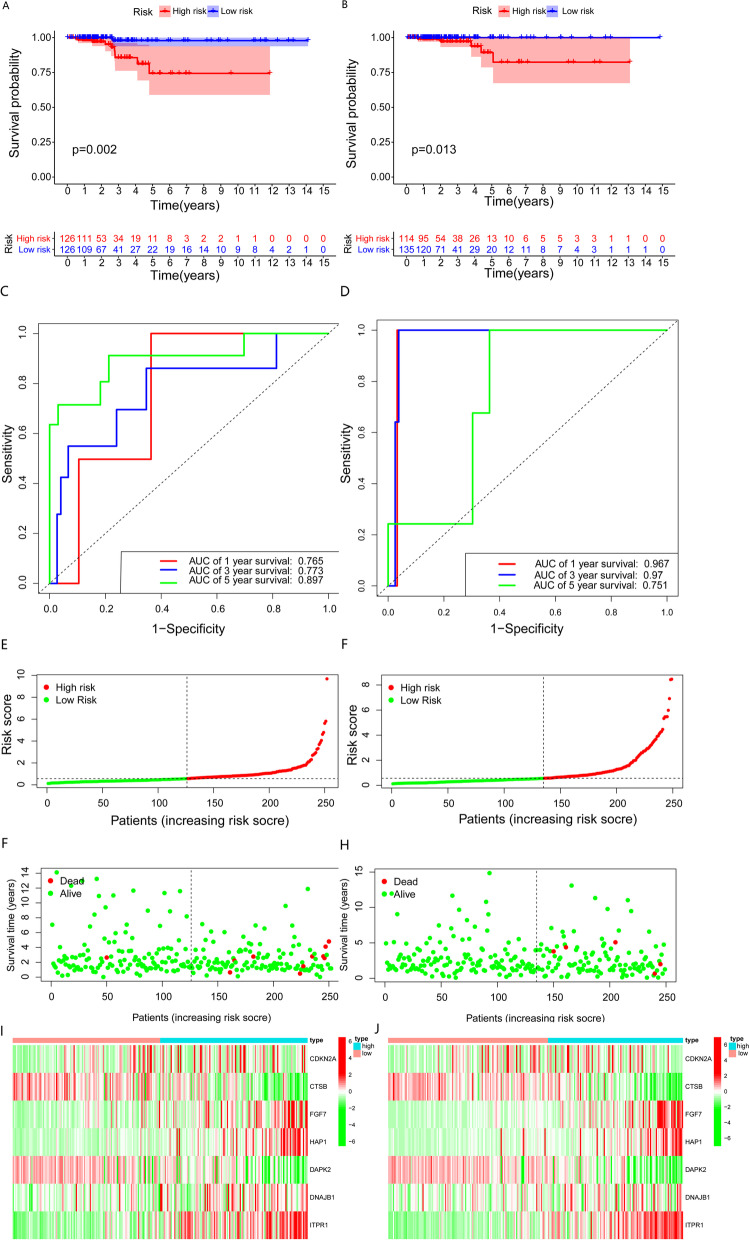
Performance and validation of the prognostic risk model: (**A**) Kaplan-Meier analysis of the training group. (**B**) Kaplan-Meier analysis of the testing group. (**C**) The ROC curve of overall survival for training group. (**D**) The ROC curve of overall survival for testing group. (**E**) The number of different risk group patients in training group. (**F**) The number of different risk group patients in testing group. (**G**) Survival status in training group. (**H**) Survival status in testing group. (**I**) Expression level of risk prognostic model gene in the training group. (**J**) Expression level of risk prognostic model gene in the testing group

Univariate Cox regression analysis of the correlations of ARGs with OS in THCA patients identified 10 prognosis-related genes (*p* < 0.05, Fig. 4A). LASSO-penalized Cox regression analysis was performed to establish a risk score prognostic model that included 7-genes (Fig. 4B, C). The risk score for each patient was determined using the equation: Risk score = (0.46026029*CDKN2A expression) − (0.29526375*CTSB expression) + (0.36900263*FGF7 expression) + (0.36720186*HAP1 expression) − (0.03896433*DAPK2 expression) + (0.20590309*DNAJB1 expression) + (0.46793624*ITPR1 expression) and median risk score was set as the cut-off value point to divide the training group into a high-risk group (*n* = 126) and low-risk group (*n* = 126). Results of the Kaplan-Meier analysis confirmed that patients with high-risk scores had poorer prognosis (*p* = 0.002, Fig. 5A). ROC analysis of the training group revealed AUC values for 1-, 3-, and 5-year OS of 0.765, 0.773, and 0.897, respectively (Fig. 5C). Additionally, THCA patients in the training group were ranked by risk score to reveal the relationship between risk score and prognosis. This analysis showed that most of the dead patients were from the high-risk group and that higher risk scores correlated with increasing mortality rate (Fig. 5E, G, I). To validate the prognostic model, similar analyses were done on the testing group and testing group on the basis of the risk score and the same cut-off criteria used for analysis in the training group (Fig. 5F, H, J). Consistent with the training group, patients with high-risk scores showed poorer prognosis than those with low scores (*p* = 0.013, Fig. 5B). The ROC curve AUC values of the testing group were 0.967, 0.97, and 0.751 for 1-, 3-, and 5-year survival (Fig. 5D). Together, these data show that the prognostic model has good predictive accuracy.

### Independent prognostic testing of the prognostic model

**Fig. 6:**
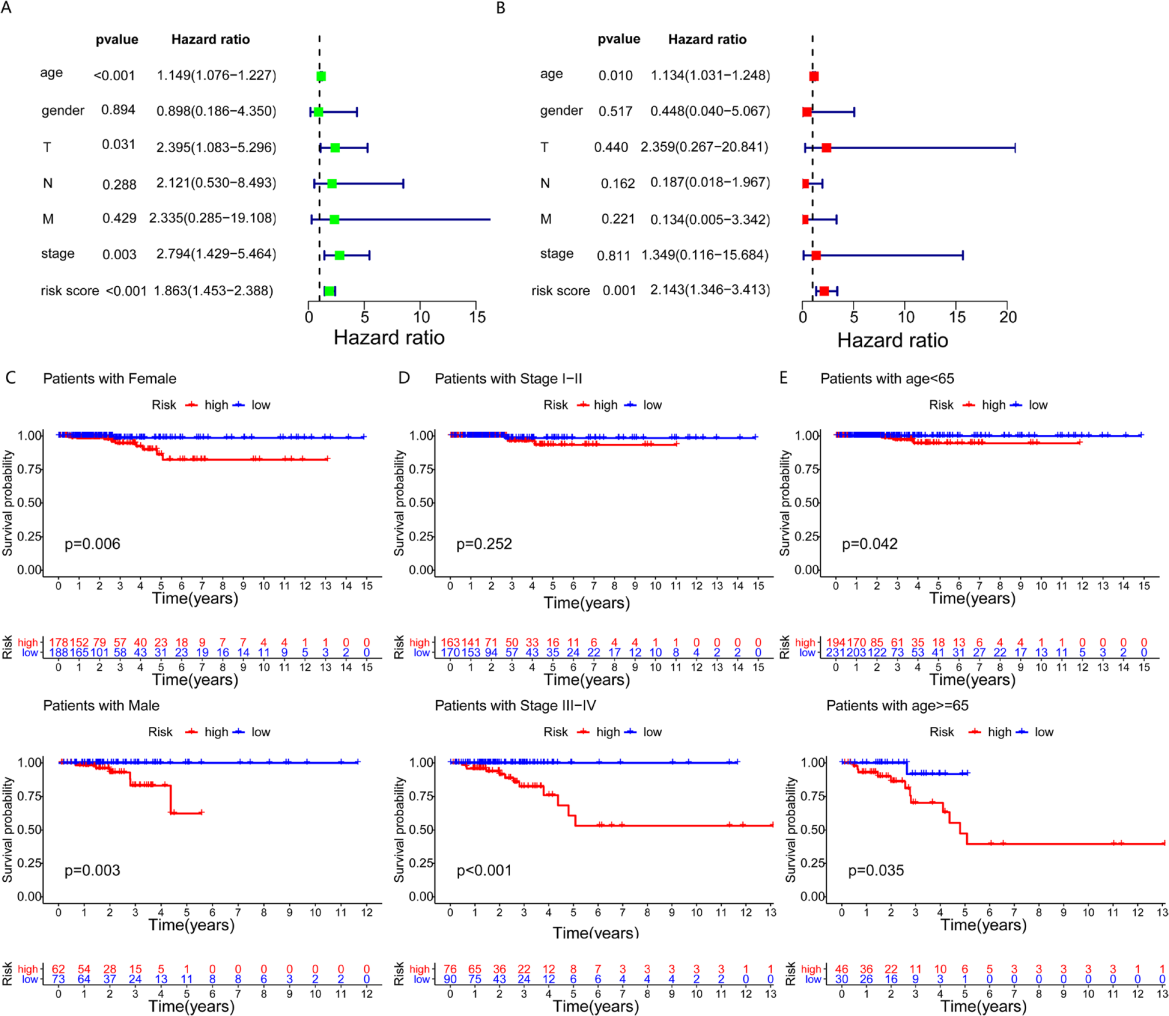
Independent prognostic testing and clinical subgroup analysis of the prognostic model: (**A**, **B**) Univariate and multivariate Cox regression analysis of clinical characteristic and risk score. (**C**) Gender. (**D**) Tumour stage. (**E**) Age

Using univariate and multivariate Cox regression analysis, we assessed whether clinical features such as age, gender, tumour stage, N (lymph node), T (tumour), M (metastasis) (Table S2), as well as the risk score prognostic model independently correlated with OS. Univariate regression analysis found that age, tumour stage, T, and risk score influenced the OS. Multivariate regression analysis revealed that age (*p* = 0.01, HR = 1.134 95%, CI=1.031–1.248) and risk score (*p* = 0.001, HR = 2.143 95% CI = 1.346–3.413) were independent risk factors for THCA prognosis (Fig. 6A, B). Clinical subgroup analysis with the prognostic model showed that prognosis was poorer in the high-risk group for both female (*p* = 0.006) and male (*p* = 0.003), disease stage III–IV (*p* < 0.001), age < 65 group (*p* = 0.042), and age ≥ 65 group (*p* = 0.035) (Fig. 6C–E). These data indicate that the model can be applied to predict prognosis independently.

### Comparison between ARG signature and TNM staging system

**Fig. 7:**
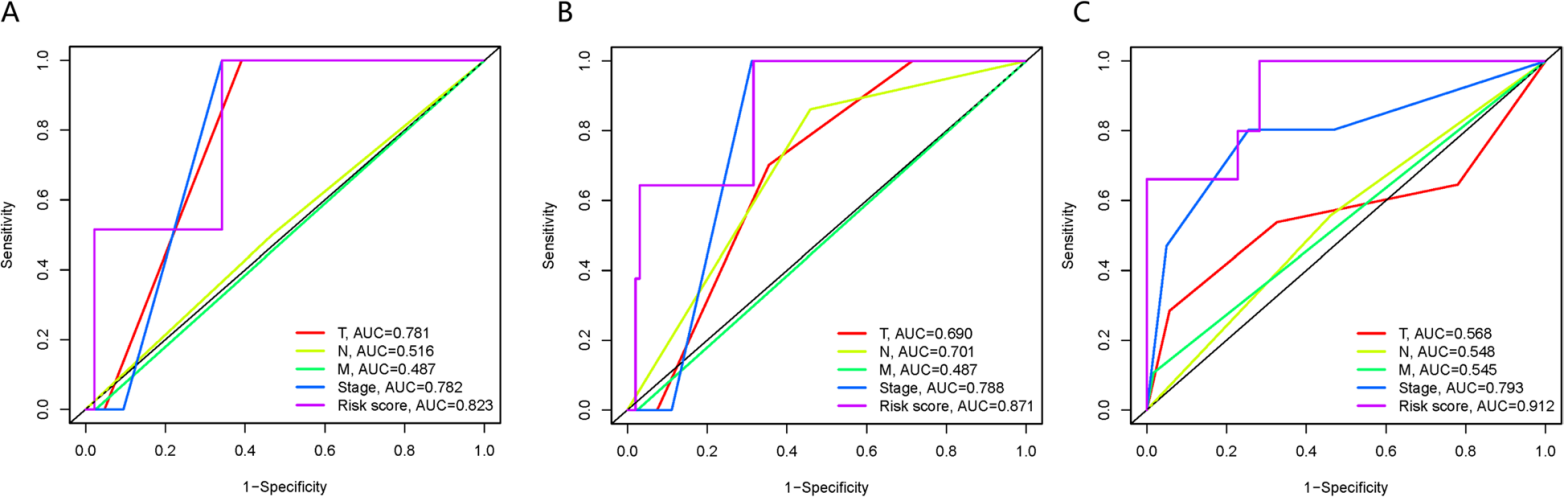
Multivariate ROC analysis compare the predictive ability of ARG signature to TNM staging system: (**A**) Multivariate ROC curves for 1-year OS. (**B**) Multivariate ROC curves for 3-year OS. (**C**) Multivariate ROC curves for 5-year OS

We plotted the ROC curves to compare the ARG signature and TNM staging system in the prediction of thyroid cancer prognosis. The ROC curves’ AUC values of ARG signature for 1-, 3-, and 5-year OS were 0.823 (Fig. 7A), 0.871 (Fig. 7B), and 0.912 (Fig. 7C) higher than the AUC values of the TNM staging system. Multivariate ROC analysis revealed that our ARG signature displayed high prognostic value compared with the TNM staging system.

### Construction and validation of the nomogram

**Fig. 8:**
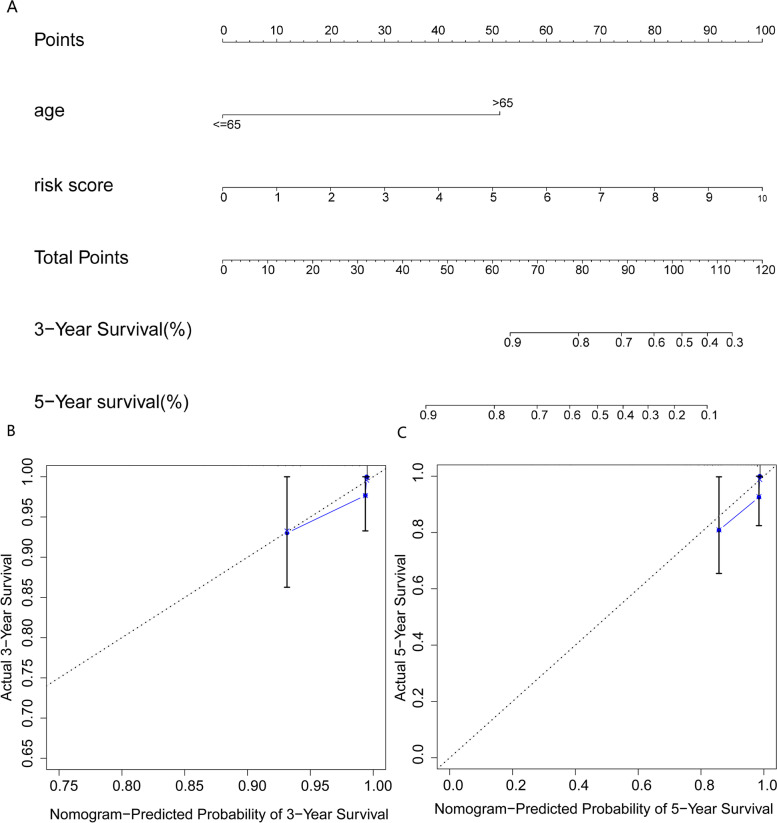
Nomogram and calibration curve of prognostic risk model: (**A**) Nomogram to predict 3- and 5-year overall survival of thyroid cancer (THCA) patients. (**B**, **C**) The calibration curves for 3-year (**B**) and 5-year (**C**) survival

To make the prognostic model convenient for clinicians, we quantified it and included other independent prognostic factors to develop a nomogram. The nomogram for 3- and 5-year OS is shown in Fig. 8A. The nomogram’s concordance index (*C*-index) of 0.911 (se = 0.044) showed that the nomogram’s predictive ability was moderate (Fig. 8B, C).

### Pathways associated with the prognostic genes

**Fig. 9:**
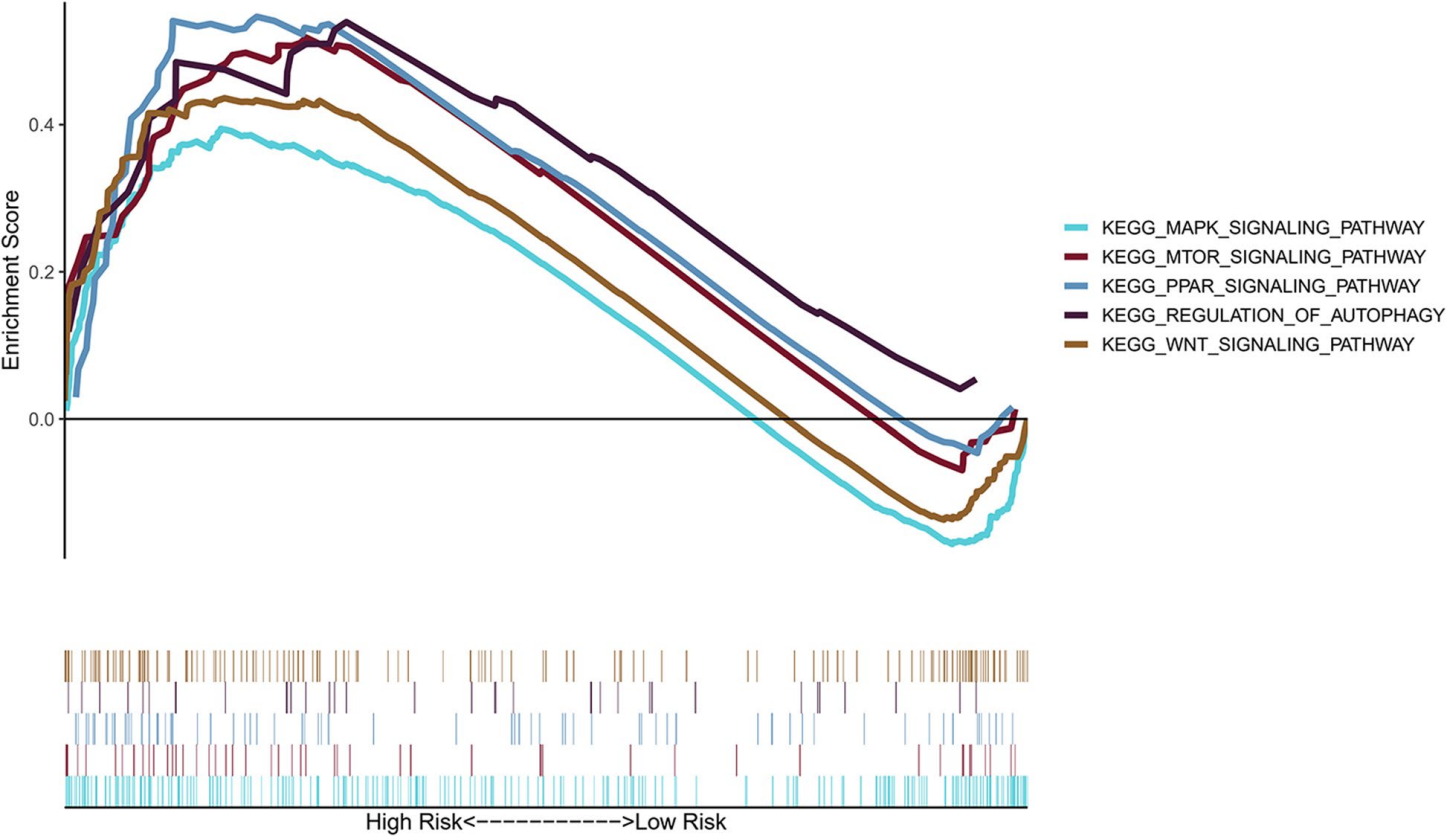
Gene set enrichment analysis

In the high-risk group, the genes were linked to autophagy and cancer pathways, including MAPK, MTOR, PPAR, regulation of autophagy, and WNT signalling (Fig. 9).

### Expression level of signature mRNAs in the thyroid cancer cell

**Fig. 10:**
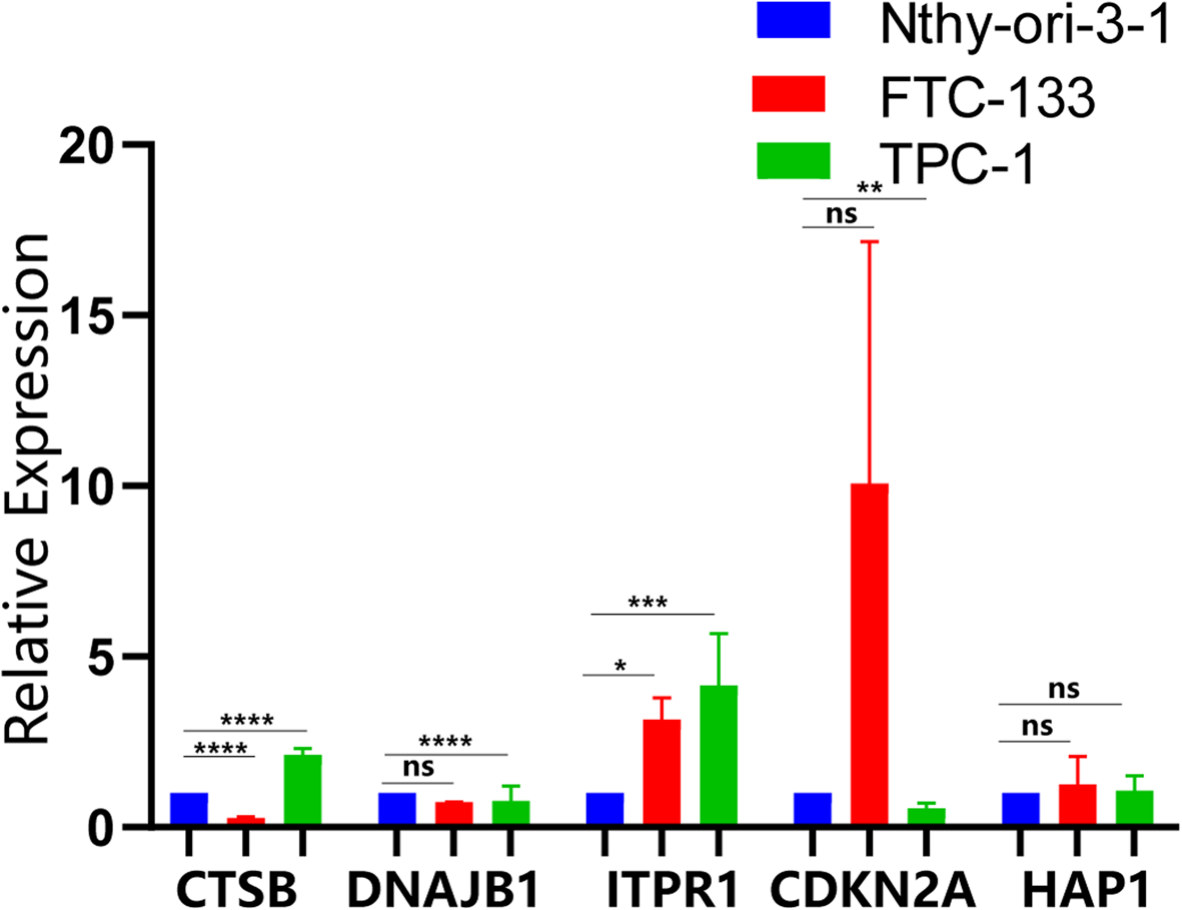
RT-qPCR analysis for the signature mRNAs. (ns: not significant, *p < 0.05, **:*p* < 0.01, ***p < 0.001, ****p < 0.0001)

We performed qRT-PCR to validate the signature mRNA expression level. The results showed that ITPR1 was highly expressed in both FTC-133 and TPC-1 thyroid cancer cell lines, and the hazard ratio of ITPR1 indicated that ITPR1 was an oncogene, and showed research potential of its biological mechanism. The expression of some mRNAs (FGF7, DAPK2) remains unknown due to missing primer sequence (Fig. 10).

## Discussion

Rapid advances in sequencing technology have led to the development of molecular markers to facilitate the detection and monitoring of the clinical course of many cancers [25]. However, the use of individual genetic markers in determining thyroid cancer prognosis has significant limitations. For example, RAS mutations, especially NRAS codon 61 mutations are associated with high incidence of distant metastasis but do not affect overall survival in THCA patients [26]. A study involving 599 PTC patients found that although the BRAF V600E mutation significantly increases PTC recurrence, it is not suitable as an independent prognostic factor [27]. Thus, an effective, gene-based independent prognostic model is needed to guide personalized therapy. Numerous studies have implicated that autophagy is crucial for the tumour progression and efficacy of targeted therapy in thyroid carcinoma. Thus, we assume autophagy-related genes have potential prognostic value in DTHCA [28–30]. To confirm our hypothesis, a multiple-step bioinformatic analysis was performed. First, we acquired autophagy-related genes from the HADb database and GO_AUTOPHAGY gene datasets. Differential expression analysis and PPI network analysis were performed to identify the hub autophagy-related genes in DTHCA. Next, we used hub autophagy-related genes to screen out the autophagy-related genes associated with prognosis by univariate Cox regression analysis and constructed a seven-autophagy-related gene signature to predict DTHCA patients’ prognosis through LASSO-penalized Cox regression analysis.

Here, we constructed a prognostic model for DTHCA comprising CDKN2A, FGF7, CTSB, HAP1, DAPK2, DNAJB1, and ITPR1 genes. CDKN2A, also known as multiple tumour suppressor 1, is an inhibitor of CDK4 that suppresses cancer cell proliferation by arresting the cell cycle at phase G1 [31–33]. However, CDKN2A upregulation may enhance autophagy in non-endometrioid endometrial carcinoma, which protects cells against unfavourable conditions, promoting tumorigenesis [34]. A previous study revealed that CDKN2A CpG islands’ methylation results in the inactivation of the CDKN2A gene, the phenomenon of hyper-methylation was more present in the PTC patients with metastases and high AMES risk [35]. This research result indicated CDKN2A might function as a tumour suppressor in PTC, but the autophagy mediated by CDKN2A effect on the thyroid has not been reported until now. FGF7, a member of the FGF family, exerts biological functions by interacting with its high-affinity binding receptor, FGFR2-IIIb [36]. The binding of FGF7 and FGFR2-IIIb could signal to SRC tyrosine kinase inducing phosphorylation of F-actin binding protein and cortactin, which promote the migration of tumour cells [37]. In our bioinformatics analysis, FGF7 is lowly expressed in thyroid cancer; Tetsuo et al. demonstrated that this expression is associated with its DNA promoter methylation [38]. It has been reported that in thyroid epithelial cells, FGFR2-IIIb reduces the expression of FGF7 and increases the ratio of FGF4/FGF7 and couples epithelial signalling with expansion of stomal to favour tumour growth [39]. DNAJB1, which belongs to the heat shock 40 protein family is associated with autophagy and participates in tumour progression [40]. In autophagy, DNAJB1 acts as a link between WIPI2 and ATG2A and WIPI2 depletion reduces the autophagosome number [41]. In addition, DNAJB1 is correlated to the essential tumour suppressor gene *p53*. Cui et al. found that DNAJB1 interacts with PDCD5 and can facilitate tumour progression via inhibiting the apoptotic function of wtp53 [42]. DAPK2 is located on chromosome 12p11.21 and encodes a calcium/calmodulin (Ca^2+^/CaM) dependent protein kinase [43]. Beclin-1, a core autophagic protein is a direct DAPK2 substrate. Activated DAPK2 induces autophagy via Beclin-1 phosphorylation [44]. 2,3′,4,4′,5-pentachlorobiphenyl (PCB118) enhances autophagy and damages thyroid ultrastructure via the DAPK2/PKD/VPS34 pathway [45]. Jiang et al. revealed that DAPK2 could promote I-κBα degradation through inducing selective autophagy and further contributing to anaplastic thyroid carcinoma development [46]. The effect of DAPK2 on differentiated thyroid tumours has remained to be reported. HAP1, encoding the Huntington (HTT) gene, is expressed in the nervous system and endocrine organs, such as the thyroid gland [47]. Several studies have shown that HAP1 function as an autophagy-regulating gene and plays a role in the development of neurodegenerative diseases and neurodevelopmental disorders [48, 49]. Diagnostic and therapeutic potential has been reported for HAP1 in breast and pancreatic cancer, but its role in thyroid cancer needs further study [50, 51]. In mammals, ITPR1 participate in encoding IP3-gated calcium channel, regulating calcium homeostasis in endoplasmic reticulum [52]. ITPR1-mediated induction of autophagy is thought to promote renal cell carcinoma by suppressing NK cells [53]. In papillary thyroid carcinoma, expression of ITPR1 was promoted via effect of lncRNA SLC26A4-AS1 mediated ETS1 recruitment, suppressing tumour growth by enhancing autophagy [54]. However, regression analysis revealed high ITPR1 expression is associated with poor THCA prognosis. CTSB belongs to the cysteine protease family that affects tumour growth and invasion through interaction with cellular proteins [55]. CTSB upregulation in papillary thyroid cancer correlates with positive lymph node metastasis and cancer cell migration via p38-mediated EMT [56]. CTSB regulates autophagy via enhancing the lysosomal membrane permeabilization process and reducing the functional lysosomes required by autophagy flux [57]. High CTSB expression has been associated with suberoylanilide hydroxamic acid (SAHA)-induced autophagy, which suppresses breast cancer growth [58]. This finding is consistent with our past findings and shows that the role of CTSB in thyroid cancer merits further investigation. Several analyses suggested that our prognostic model has excellent accuracy and independence. Our prognostic model’s predictive ability for DTHCA patient’s OS is better than the widely used TNM staging system by conducting multivariate ROC analysis. However, this result still needs more patients to validate in the clinical practice. After that, a nomogram was constructed to screen out the relatively high-risk patients in clinical practice; and this population might need extended resection and lymphadenectomy. Nomograms are widely used to predict prognosis, skip metastasis, and central lymph node metastasis in thyroid carcinoma [59–61]. To give clinicians a practical tool for individualized prognosis prediction, we constructed a nomogram based on the independent factors in the multivariate Cox regression analysis. It is worth mentioning that our nomogram has better predictive ability than other thyroid prognosis-related nomograms, as quantified by the concordance index (*C*-index:0.911 95% CI:0.867–0.955), the *C*-index of Wang’s nomogram was 0.797 (95% CI: 0.730–0.864), and that of Zhang’s nomogram was 0.717 (95% CI, 0.603–0.831) [62, 63].

However, this study had two main limitations. First, all transcriptome data and information on clinical characteristics were obtained from public datasets and lacked external validation. Second, further clinical cases and experiments are needed to clarify the mechanism by which ARGs affect thyroid cancer development. Despite these limitations, our prognostic model showed excellent predictive accuracy and clinical applicability and may guide individualized therapy.

## Conclusion

In conclusion, we developed a novel seven-mRNA (CDKN2A, FGF7, CTSB, HAP1, DAPK2, DNAJB1, ITPR1) risk model and nomogram that could help assess the prognosis of patients and guide clinical decision-making and individualized therapy for differentiated thyroid cancer.

## Supplementary Information

**Additional file 1.**

**Additional file 2.**

**Additional file 3.**

**Additional file 4.**

## Data Availability

The data that support the findings of this study are available in The Cancer Genome Atlas databases at https://www.cancer.gov/about-nci/organization/ccg/research/structural-genomics/tcga, reference number: TCGA-THCA.

## Abbreviations

OS: Overall survival
THCA: Thyroid cancer
DTHCA: Differentiated thyroid carcinoma
AUC: Area under ROC curve
ARGs: Autophagy-related genes
TCGA: The Cancer Genome Atlas
PPI: Protein-protein interaction
FDR: False discovery rate
GO: Gene Ontology
KEGG: Kyoto Encyclopedia of Genes and Genomes
HR: Hazard ratio
GSEA: Gene set enrichment analysis
RAI: Radioiodine
NIS: Sodium iodide symporter

## References

[1] Sun T, Li X, Zhang P, Chen WD, Zhang HL, Li DD, Deng R, Qian XJ, Jiao L, Ji J, Li YT, Wu RY, Yu Y, Feng GK, Zhu XF (2015). Acetylation of Beclin 1 inhibits autophagosome maturation and promotes tumor growth. Nat. Commun..

[2] Xie Y, Kang R, Sun X, Zhong M, Huang J, Klionsky DJ, Tang D (2015). Posttranslational modification of autophagy-related proteins in macroautophagy. Autophagy.

[3] Feng Y, He D, Yao Z, Klionsky DJ (2014). The machinery of macroautophagy. Cell Res.

[4] Onorati AV, Dyczynski M, Ojha R, Amaravadi RK (2018). Targeting autophagy in cancer. Cancer.

[5] Nassour J, Radford R, Correia A, Fusté JM, Schoell B, Jauch A, Shaw RJ, Karlseder J (2019). Autophagic cell death restricts chromosomal instability during replicative crisis. Nature.

[6] Dower CM, Wills CA, Frisch SM, Wang HG (2018). Mechanisms and context underlying the role of autophagy in cancer metastasis. Autophagy.

[7] Pecoraro A, Carotenuto P, Franco B, De Cegli R, Russo G, Russo A (2020). Role of uL3 in the crosstalk between nucleolar stress and autophagy in colon cancer cells. Int. J. Mol. Sci..

[8] Siegel RL, Miller KD, Fuchs HE, Jemal A (2021). Cancer Statistics, 2021. Cancer J Clin.

[9] Kitahara CM, Sosa JA (2020). Understanding the ever-changing incidence of thyroid cancer. Nature reviews. Endocrinol.

[10] Lim H, Devesa SS, Sosa JA, Check D, Kitahara CM (2017). Trends in thyroid cancer incidence and mortality in the United States, 1974-2013. JAMA.

[11] Li M, Dal Maso L, Vaccarella S (2020). Global trends in thyroid cancer incidence and the impact of overdiagnosis. Lancet Diabetes Endocrinol..

[12] Hong S, Won YJ, Park YR, Jung KW, Kong HJ, Lee ES, Community of Population-Based Regional Cancer Registries (2020). Cancer statistics in Korea: incidence, mortality, survival, and prevalence in 2017. Cancer Res. Treat..

[13] Asa SL (2019). The current histologic classification of thyroid cancer. Endocrinol. Metab. Clin. North Am..

[14] Yan KL, Li S, Tseng CH, Kim J, Nguyen DT, Dawood NB, Livhits MJ, Yeh MW, Leung AM. Rising incidence and incidence-based mortality of thyroid cancer in California, 2000-2017. J Clin Endocrinol Metab. 2020;105(6):dgaa121. 10.1210/clinem/dgaa121 10.1210/clinem/dgaa121 32166320

[15] Ibrahim EY, Busaidy NL (2017). Treatment and surveillance of advanced, metastatic iodine-resistant differentiated thyroid cancer. Curr. Opin. Oncol.

[16] Kreissl MC, Janssen M, Nagarajah J (2019). Current treatment strategies in metastasized differentiated thyroid cancer. J Nuclear Med.

[17] Jin Y, Van Nostrand D, Cheng L, Liu M, Chen L (2018). Radioiodine refractory differentiated thyroid cancer. Crit. Rev. Oncol. Hematol..

[18] Chereau N, Oyekunle TO, Zambeli-Ljepović A, Kazaure HS, Roman SA, Menegaux F, Sosa JA (2019). Predicting recurrence of papillary thyroid cancer using the eighth edition of the AJCC/UICC staging system. Br. J. Surg..

[19] Hou X, Shi X, Zhang W, Li D, Hu L, Yang J, Zhao J, Wei S, Wei X, Ruan X, Zheng X, Gao M (2021). LDHA induces EMT gene transcription and regulates autophagy to promote the metastasis and tumorigenesis of papillary thyroid carcinoma. Cell Death Dis.

[20] Wang M, Qiu S, Qin J (2019). Baicalein induced apoptosis and autophagy of undifferentiated thyroid cancer cells by the ERK/PI3K/Akt pathway. Am. J. Transl. Res.

[21] Oh JM, Ahn BC (2021). Molecular mechanisms of radioactive iodine refractoriness in differentiated thyroid cancer: impaired sodium iodide symporter (NIS) expression owing to altered signaling pathway activity and intracellular localization of NIS. Theranostics.

[22] Tesselaar MH, Crezee T, Swarts HG, Gerrits D, Boerman OC, Koenderink JB, Stunnenberg HG, Netea MG, Smit JW, Netea-Maier RT, Plantinga TS (2017). Digitalis-like compounds facilitate non-medullary thyroid cancer redifferentiation through intracellular Ca2+, FOS, and autophagy-dependent pathways. Mol. Cancer Ther.

[23] Wang W, Kang H, Zhao Y, Min I, Wyrwas B, Moore M, Teng L, Zarnegar R, Jiang X, Fahey TJ (2017). Targeting autophagy sensitizes BRAF-mutant thyroid cancer to vemurafenib. J. Clin. Endocrinol. Metab..

[24] Wang Y, Zhao W, Xiao Z, Guan G, Liu X, Zhuang M (2020). A risk signature with four autophagy-related genes for predicting survival of glioblastoma multiforme. J. Cell. Mol. Med..

[25] Jayanthi V, Das AB, Saxena U (2017). Recent advances in biosensor development for the detection of cancer biomarkers. Biosens Bioelectron.

[26] Jang EK, Song DE, Sim SY, Kwon H, Choi YM, Jeon MJ, Han JM, Kim WG, Kim TY, Shong YK, Kim WB (2014). NRAS codon 61 mutation is associated with distant metastasis in patients with follicular thyroid carcinoma. Thyroid.

[27] Enumah S, Fingeret A, Parangi S, Dias-Santagata D, Sadow PM, Lubitz CC (2020). BRAF^V600E^ mutation is associated with an increased risk of papillary thyroid cancer recurrence. World J Surg.

[28] Zhou H, Xie X, Chen Y, Lin Y, Cai Z, Ding L, Wu Y, Peng Y, Tang S, Xu H. Chaperone-mediated autophagy governs progression of papillary thyroid carcinoma via PPARγ-SDF1/CXCR4 signaling. J Clin Endocrinol Metabol. 2020;105(10):dgaa366. 10.1210/clinem/dgaa366 10.1210/clinem/dgaa366 32556197

[29] Yi H, Ye T, Ge M, Yang M, Zhang L, Jin S, Ye X, Long B, Li L (2018). Inhibition of autophagy enhances the targeted therapeutic effect of sorafenib in thyroid cancer. Oncol Rep.

[30] Liu K, Yu Q, Li H, Xie C, Wu Y, Ma D, Sheng P, Dai W, Jiang H (2020). BIRC7 promotes epithelial-mesenchymal transition and metastasis in papillary thyroid carcinoma through restraining autophagy. Am J Cancer Res.

[31] Mao X, Li B, Liang Y, Li S, Zhou J, He Q, Jiang N, Chen Y, Sun Y, Cui Y, Jiang W, Wang H, Wang L, Ke Z (2018). Auxiliary diagnostic value of p16 amplification combined with the detection of heterozygous and homozygous loss for urothelial carcinoma. Oncol Lett.

[32] Serrano M (1997). The tumor suppressor protein p16INK4a. Experiment Cell Rese.

[33] Appay R, Dehais C, Maurage CA, Alentorn A, Carpentier C, Colin C, Ducray F, Escande F, Idbaih A, Kamoun A, Marie Y, Mokhtari K, Tabouret E, Trabelsi N, Uro-Coste E, Delattre JY, Figarella-Branger D, Network POLA (2019). CDKN2A homozygous deletion is a strong adverse prognosis factor in diffuse malignant IDH-mutant gliomas. Neuro-Oncol.

[34] Devis-Jauregui L, Eritja N, Davis ML, Matias-Guiu X, Llobet-Navàs D (2021). Autophagy in the physiological endometrium and cancer. Autophagy.

[35] Wang P, Pei R, Lu Z, Rao X, Liu B (2013). Methylation of p16 CpG islands correlated with metastasis and aggressiveness in papillary thyroid carcinoma. J Chin Med Assoc.

[36] Lezmi G, Verkarre V, Khen-Dunlop N, Vibhushan S, Hadchouel A, Rambaud C, Copin MC, Rittie JL, Benachi A, Fournet JC, Delacourt C (2013). FGF10 Signaling differences between type I pleuropulmonary blastoma and congenital cystic adenomatoid malformation. Orphanet J Rare Dis.

[37] Belleudi F, Scrofani C, Torrisi MR, Mancini P (2011). Polarized endocytosis of the keratinocyte growth factor receptor in migrating cells: role of SRC-signaling and cortactin. PloS one.

[38] Kondo T, Zhu X, Asa SL, Ezzat S (2007). The cancer/testis antigen melanoma-associated antigen-A3/A6 is a novel target of fibroblast growth factor receptor 2-IIIb through histone H3 modifications in thyroid cancer. Clin Cancer Res.

[39] Guo M, Liu W, Serra S, Asa SL, Ezzat S (2012). FGFR2 isoforms support epithelial-stromal interactions in thyroid cancer progression. Cancer Res.

[40] Ren H, Luo M, Chen J, Zhou Y, Li X, Zhan Y, Shen D, Chen B (2019). Identification of TPD52 and DNAJB1 as two novel bile biomarkers for cholangiocarcinoma by iTRAQ-based quantitative proteomics analysis. Oncol Rep.

[41] Behrends C, Sowa ME, Gygi SP, Harper JW (2010). Network organization of the human autophagy system. Nature.

[42] Cui X, Choi HK, Choi YS, Park SY, Sung GJ, Lee YH, Lee J, Jun WJ, Kim K, Choi KC, Yoon HG (2015). DNAJB1 destabilizes PDCD5 to suppress p53-mediated apoptosis. Cancer Lett.

[43] Kawai T, Nomura F, Hoshino K, Copeland NG, Gilbert DJ, Jenkins NA, Akira S (1999). Death-associated protein kinase 2 is a new calcium/calmodulin-dependent protein kinase that signals apoptosis through its catalytic activity. Oncogene.

[44] Shiloh R, Gilad Y, Ber Y, Eisenstein M, Aweida D, Bialik S, Cohen S, Kimchi A (2018). Non-canonical activation of DAPK2 by AMPK constitutes a new pathway linking metabolic stress to autophagy. Nature Commun.

[45] Zhou Q, Wang L, Chen H, Xu B, Xu W, Sheng Y, Duan Y (2019). 2,3′,4,4′,5-Pentachlorobiphenyl induced autophagy of the thyrocytes via DAPK2/PKD/VPS34 pathway. Arch Toxicol.

[46] Jiang Y, Liu J, Xu H, Zhou X, He L, Zhu C (2021). DAPK2 activates NF-κB through autophagy-dependent degradation of I-κBα during thyroid cancer development and progression. Ann Transl Med.

[47] Liao M, Shen J, Zhang Y, Li SH, Li XJ, Li H (2005). Immunohistochemical localization of huntingtin-associated protein 1 in endocrine system of the rat. J Histochem Cytochem.

[48] Wang T, Wang J, Wang J, Mao L, Tang B, Vanderklish PW, Liao X, Xiong ZQ, Liao L. HAP1 is an in vivo UBE3A target that augments autophagy in a mouse model of Angelman syndrome. Neurobiol Dis. 2019;132:104585. 10.1016/j.nbd.2019.104585. 10.1016/j.nbd.2019.104585 31445164

[49] Wong YC, Holzbaur EL (2014). The regulation of autophagosome dynamics by huntingtin and HAP1 is disrupted by expression of mutant huntingtin, leading to defective cargo degradation. J Neurosci..

[50] Zhu L, Song X, Tang J, Wu J, Ma R, Cao H, Ji M, Jing C, Wang Z (2013). Huntingtin-associated protein 1: a potential biomarker of breast cancer. Oncol Rep.

[51] Kakolyris S, Kaklamanis L, Giatromanolaki A, Koukourakis M, Hickson ID, Barzilay G, Turley H, Leek RD, Kanavaros P, Georgoulias V, Gatter KC, Harris AL (1998). Expression and subcellular localization of human AP endonuclease 1 (HAP1/Ref-1) protein: a basis for its role in human disease. Histopathol.

[52] Roy D, Sin SH, Damania B, Dittmer DP (2011). Tumor suppressor genes FHIT and WWOX are deleted in primary effusion lymphoma (PEL) cell lines. Blood.

[53] Messai Y, Noman MZ, Hasmim M, Janji B, Tittarelli A, Boutet M, Baud V, Viry E, Billot K, Nanbakhsh A, Ben Safta T, Richon C, Ferlicot S, Donnadieu E, Couve S, Gardie B, Orlanducci F, Albiges L, Thiery J, Olive D (2014). ITPR1 protects renal cancer cells against natural killer cells by inducing autophagy. Cancer Res.

[54] Peng D, Li W, Zhang B, Liu X (2021). Overexpression of lncRNA SLC26A4-AS1 inhibits papillary thyroid carcinoma progression through recruiting ETS1 to promote ITPR1-mediated autophagy. J. Cell. Mol. Med.

[55] Mijanović O, Branković A, Panin AN (2019). Cathepsin B: A sellsword of cancer progression. Cancer Lett..

[56] Kim EK, Song MJ, Jang HH, Chung YS (2020). Clinicopathologic analysis of cathepsin B as a prognostic marker of thyroid cancer. Int. J. Mol. Sci..

[57] You L, Wang Z, Li H, Shou J, Jing Z, Xie J, Sui X, Pan H, Han W (2015). The role of STAT3 in autophagy. Autophagy.

[58] Han H, Zhou H, Li J, Feng X, Zou D, Zhou W (2017). TRAIL DR5-CTSB crosstalk participates in breast cancer autophagy initiated by SAHA. Cell Death Dis.

[59] Zhang L, Wang Y, Li X, Wang Y, Wu K, Wu J, Liu Y (2020). Identification of a recurrence signature and validation of cell infiltration level of thyroid cancer microenvironment. Front Endocrinol.

[60] Wang W, Yang Z, Ouyang Q (2020). A nomogram to predict skip metastasis in papillary thyroid cancer. World J Surg Oncol.

[61] Feng JW, Hong LZ, Wang F, Wu WX, Hu J, Liu SY, Jiang Y, Ye J (2021). A nomogram based on clinical and ultrasound characteristics to predict central lymph node metastasis of papillary thyroid carcinoma. Front Endocrinol.

[62] Ruchong P, Haiping T, Xiang W (2021). A five-gene prognostic nomogram predicting disease-free survival of differentiated thyroid cancer. Dis Markers.

[63] Tang J, Jiang S, Gao Q, Xi X, Gao L, Zhao R, Lai X, Zhang B, Jiang Y (2021). Development and validation of a nomogram based on stromal score to predict progression-free survival of patients with papillary thyroid carcinoma. Cancer Med.

